# Considerations of prescribers and pharmacists for the use of non‐selective β‐blockers in asthma and COPD patients: An explorative study

**DOI:** 10.1111/jep.12869

**Published:** 2018-01-10

**Authors:** Esther Kuipers, Michel Wensing, Peter A.G.M. De Smet, Martina Teichert

**Affiliations:** ^1^ Department of IQ Healthcare, Radboud Institute for Health Sciences Radboud University Medical Centre Nijmegen The Netherlands; ^2^ BENU Apotheek Zeist West Zeist The Netherlands; ^3^ Department of General Practice and Health Services Research University Hospital Heidelberg Heidelberg Germany; ^4^ Department of Clinical Pharmacy, Radboud Institute for Health Sciences Radboud University Medical Centre Nijmegen The Netherlands; ^5^ Department of Clinical Pharmacy & Toxicology Leiden University Medical Centre Leiden The Netherlands

**Keywords:** asthma, COPD, inhalation, medication, pharmacotherapy, primary care

## Abstract

**Rationale, aims, and objectives:**

Despite recommendations in prevailing guidelines to avoid the use of non‐selective (NS) β‐blockers in patients with asthma or COPD, on average, 10 patients per community pharmacy receive NS β‐blockers monthly. The aim of our study was to identify the reasons of prescribers and pharmacists to treat asthma and COPD patients with NS β‐blockers.

**Methods:**

Fifty‐three community pharmacists in the Netherlands selected patients with actual concurrent use of inhalation medication and NS β‐blockers. For at least 5 patients, each pharmacist screened all medication surveillance signals and actions taken at first dispensing. Each pharmacist selected 3 different initial prescribers for a short interview to explore their awareness of the co‐morbidity and reasons to apply NS β‐blockers.

**Results:**

Pharmacists identified 827 asthma/COPD patients with actual use of NS β‐blockers. From these, 153 NS β‐blocker prescribers were selected and interviewed (64 general practitioners, 45 ophthalmologists, 24 cardiologists, and 20 other prescribers). One hundred seven prescribers were aware of the drug‐disease interaction of the asthma or COPD co‐morbidity when initiating the NS β‐blocker, and 46 were not. From these, 40 prescribers did not consider the contraindication to be relevant.

For 299 patients, medication surveillance signals and actions at first dispensing were retrieved. Patients used predominantly ocular timolol (39.8%), and the oral preparations propranolol (30.8%) and carvedilol (15.1%). In 154 cases, the pharmacy system generated a warning alert.

**Conclusions:**

A substantial number of prescribers was unaware of the co‐morbidity or did not regard NS β‐blockers contraindicated, despite prevailing clinical guidelines. Improvement programs should target prescribers' awareness and knowledge of NS β‐blockers in patients with asthma or COPD.

## 1 INTRODUCTION

Treatment with β_2_‐adrenoceptor agonists plays an important role in the treatment of asthma and chronic obstructive pulmonary disease (COPD). In patients with COPD, short‐acting β_2_‐agonists (SABA) and long‐acting β_2_‐agonists (LABA) are recommended to relieve airflow limitation.^1^, ^2^ In patients with asthma, SABA are used for acute relief of symptoms and LABA are used as add‐ons to inhaled corticosteroids (ICS) for patients not achieving asthma control on therapy with ICS alone.^3^, ^4^

β_2_‐Receptors are found in smooth muscle cells of the bronchi, whereas β_1_‐adrenoceptors are mainly located in the heart.^1^, ^2^, ^3^, ^4^ Blocking β_1_‐receptors in the heart is essential in the treatment of several cardiovascular diseases, causing a reduction in heart rate and contraction force. Systemic treatment with β‐blockers was shown to reduce mortality and the risk of arrhythmias and to delay heart failure after a myocardial infarction.^5^, ^6^ Ocular β‐blockers are indicated for glaucoma and reduce the intraocular pressure by decreasing the production of aqueous humour.^5^, ^7^ Due to the potential risk of triggering bronchoconstriction and an insufficient response to bronchodilator therapy during an exacerbation, use of non‐selective (NS) β‐blockers in patients with asthma or COPD is contraindicated according to prevailing guidelines for lung diseases and glaucoma.^1^, ^2^, ^3^, ^4^, ^8^

Some β‐blockers are selective antagonists at the β1‐receptor (eg, atenolol, bisoprolol, and metoprolol), usually called “cardioselective” β‐blockers. Other β‐blockers (eg, propranolol and timolol) also show antagonist activity at β2‐adrenoceptors; these drugs are referred to as “non‐selective” β‐blockers. Receptor selectivity of β‐blockers is a dose‐dependent property, as with increasing dose, the β_1_‐selectivity decreases.^5^, ^9^, ^10^, ^11^ Moreover, even in cardioselective β‐blockers, β1‐selectivity was shown to be relative,^12^ and these β‐blockers can also cause β_2_‐mediated respiratory side effects as bronchospasm or a fall in forced expiratory volume in 1 second in susceptible individuals.^5^, ^13^, ^14^, ^15^, ^16^ However, the contraindication in the guidelines is limited to NS β‐blockers; fewer hospitalizations and emergency department visits occurred with cardioselective β‐blockers, compared to NS β‐blockers.^5^, ^17^, ^18^, ^19^ There was no convincing evidence for a clinically relevant influence of selective β‐blockers on bronchoconstriction.^1^, ^2^, ^3^, ^4^, ^5^, ^7^, ^15^, ^16^, ^20^, ^21^, ^22^, ^23^, ^24^, ^25^

Despite this contraindication, on average, 10 users of inhalation medication per community pharmacy in the Netherlands were detected with NS β‐blockers co‐medication^26^, ^27^ in 2016. From dispensing data only, it cannot be deduced whether or not the vprescribing and dispensing of NS β‐blockers for this population were due to deliberate clinical reasoning of prescribers and pharmacists.

Consequently, the primary objectives of this study were to assess whether prescribers were aware of the lung disease at the start of the NS β‐blocker and, if so, to explore the reasons why these were prescribed. Furthermore, this study aimed to examine the way of signalling this drug‐disease interaction in the pharmacy computer system and how the pharmacist dealt with this surveillance signals in daily practice.

## 2 METHODS

### 2.1 Study design

This was an explorative observational study in 53 community pharmacies in the Netherlands. The study protocol was approved by the Ethical Committee of the Radboud UMC Nijmegen (approval number: 2015‐2185).

### 2.2 Setting

Fifty‐three community pharmacists, located in different areas in the Netherlands, participated in this study between February and July 2016. These pharmacists belonged to 3 different educational groups within the national postgraduate specialization programme to become community pharmacists. Participating in research practice is part of the last year of this educational programme. Thus, the participating pharmacists were a convenience sample of all pharmacists in the Netherlands. Dutch pharmacists have a professional and legal responsibility for the drug treatment of their patients.^28^ All pharmacists in the Netherlands use a computer system, designed to signalize drug‐drug interactions and, if applicable, intolerances and drug‐disease interactions. Pharmacists use these signals to identify drug therapy‐related problems and consult prescribers.

Handling of all monitoring signals is registered in the local pharmacy system.

### 2.3 Patient selection—identification

Patients were selected from pharmacy dispensing data. In the Netherlands, all medication dispensed on prescription is registered in the local pharmacy system. Dispensing data from more than 90% of the 1981 community pharmacies in the Netherlands are delivered routinely to the Foundation of Pharmaceutical Statistics (SFK). These data provide detailed information on the drugs dispensed, including the codes from the Anatomic Therapeutic Chemical (ATC) system of the World Health Organization.^29^ The computerized pharmacy system can only calculate correct periods of drug use, if the total number of dispensed drug doses and the prescribed daily dose is entered.^30^ From these data, SFK generates periodically online reports for participating pharmacies, to detect possible medication problems and to improve pharmaceutical care.

Participating pharmacists were provided with an automated web report that identified all users of inhalation medication for their pharmacy with actual use of NS (oral or ocular) β‐blockers. The SFK web report presented all current users of inhalation medication for asthma or COPD (SABA, LABA, ICS or combinations of ICS/LABA, and short‐ and long‐acting muscarinic antagonists; ATC code R03) that were also current users of an oral NS β‐blocker (ATC codes C07AA, C07AG, C07BA, C07CA, C07DA, C07EA, and C07FA) or an ocular NS β‐blocker (ATC codes: S01ED, except S01ED02 and S01ED52), independent of which medication had been started first.

#### 2.3.1 Selection of NS β‐blocker initiators for prescriber interview

From the SFK web report, each pharmacist selected 3 prescribers from different disciplines for an interview about their choice to initiate the NS β‐blocker for this population, based on a semistructured format (Appendix A). Pharmacists were asked to look for the first dispensing of an NS β‐blocker in the patients dispensing history to identify the initial prescriber (initiator). A first dispensing was defined as the dispensing of an NS β‐blocker without any dispensing of an NS β‐blocker within the preceding 12 months. The first question in these interviews was whether the initiator was aware of the airway disease when starting the NS β‐blocker. When this was the case, the pharmacist asked for the reasons to prescribe an NS β‐blocker despite the drug‐disease interaction and whether the choice would have been reconsidered if the patient would suffer from exacerbations after the start of the NS β‐blocker. If the initiator was not aware, possible reasons for this were explored. Several possible reasons (eg, lack of a complete patient file and not interested in co‐morbidities) were prepared in a digital form, with the possibility to add additional answers.

#### 2.3.2 Selection of patients for occurrence and handling of surveillance signals

From the web report, each pharmacist selected at least 5 current users of inhalation medication in combination with an NS β‐blocker (aged 18 years or older). For each identified patient, all medication surveillance signals and actions taken at first dispensing were screened and the diagnosis of asthma or COPD and the reason for prescribing the oral NS β‐blocker were verified with the general practitioner (GP), if possible. In daily practice, when patients are treated by different health care providers, lack of communication can lead to incomplete dossiers of all professionals involved. Moreover, diagnoses and reasons for drug prescribing are often not communicated to the community pharmacist. As an approach, the pharmacy information system can generate “deduced contraindication” signals from the medication dispensed. These can be judged by the pharmacist, verified by the prescriber, and stored in the system. For example, when a patient uses antidiabetic medication, the pharmacist can enter the contraindication “diabetes mellitus” into the pharmacy information system.

### 2.4 Analysis

With descriptive analysis, answers were stratified for medical specialism (GP, cardiologist or ophthalmologist, pulmonologist, neurologists, psychiatrist, and other specialists) and awareness of the contraindication, and categorized for reasons that the NS β‐blocker was prescribed in spite of the airway disease. Differences between prescribers were examined with the chi‐square test, using IBM Corp SPSS statistics, Chicago IL, USA, version 22.

## 3 RESULTS

In 53 pharmacies, 827 patients were identified by the web report, dispensed NS β‐blocker co‐medication concomitantly with inhalation therapy for overlapping time periods. From this selection, medication surveillance signals were checked for 299 patients (Table 1) and 153 prescribers were interviewed.

**Table 1:** Characteristics of actual users of non‐selective (NS) β‐blockers together with inhalation medication (n = 299)

Male sex (n, %)	133 (44.5%)
Age, y; mean (SD)	69.52 (12.23)
Lung disease (n, %)	
Asthma	122 (40.8%)
COPD	106 (35.5%)
Asthma/COPD combined	51 (17.1%)
Other airway disease	2 (0.7%)
Unknown	18 (6.0%)
NS β‐blocker (n, %)	
Timolol ocular	133 (39.8%)
Levobunolol ocular	1 (0.33%)
Propranolol	92 (30.8%)
Carvedilol	45 (15.1%)
Sotalol	15 (5.0%)
Labetalol	13 (4.3%)
Indication NS β‐blocker (n, %)	
Glaucoma	130 (43.5%)
Hypertension	45 (15.1%)
Heart failure	24 (8.0%)
Angina pectoris	16 (5.4%)
Prophylaxis migraine	15 (5.0%)
Atrial fibrillation	4 (1.3%)
Anxiety	4 (1.3%)
Tremor	4 (1.3%)
Other indications	7 (2.3%)
Unknown	50 (16.7%)

Abbreviation: COPD, chronic obstructive pulmonary disease.

### 3.1 Prescribers—interviews

A convenience sample of 153 NS β‐blocker initiators (for 3 patients per pharmacy) was interviewed for this study. Sixty‐four initiators were GPs, 45 were ophthalmologists, 24 cardiologists, and 20 other prescribers (eg, neurologists, psychiatrists, and doctors of internal medicine).

**Figure 1:**
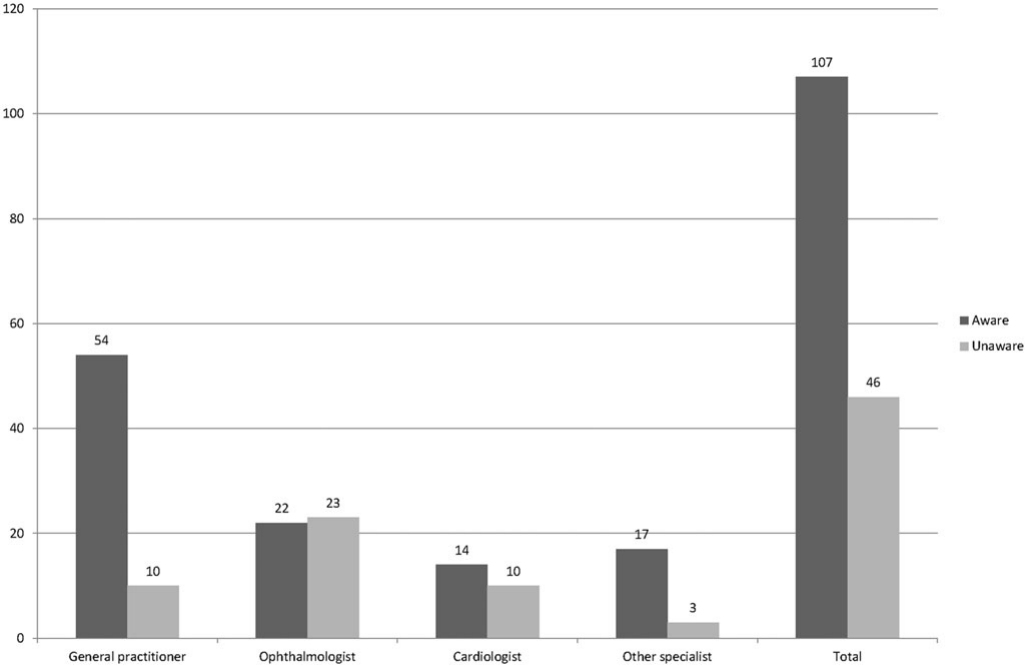
Prescribers' awareness of asthma or chronic obstructive pulmonary disease co‐morbidity at the moment of prescribing non‐selective β‐blockers

One hundred seven initiators (69.9%) indicated to have been aware of the drug‐disease interaction at the moment of prescribing the NS β‐blocker (Figure 1). Reasons for choosing the NS β‐blocker, despite the drug‐disease interaction, are shown in Table 2. Of all initiators, 40 (37.4%) considered the co‐morbidity asthma or COPD to be not relevant. Stratification for the different disciplines showed that 38.9% of the GPs, 63.6% of the ophthalmologists, and 35.7% of the interviewed cardiologists had this opinion (*P* = .032). During the interviews, ophthalmologists mentioned regularly to have never seen exacerbations in daily practice. Twenty‐five initiators indicated a lack of alternative medication: 15 GPs (27.8%), 4 cardiologists (28.6%), 1 ophthalmologist (4.5%), and 5 of other disciplines (29.4%, *P* = .139).

**Table 2:** Prescribers' reasons for choosing the non‐selective (NS) β‐blocker despite awareness of the contraindication

Statements	GP (n = 54)	OP (n = 22)	CA (n = 14)	OS (n = 17)	Total (n = 107)
Contraindication not regarded to be relevant	21 (38.9%)	14 (63.6%)	5 (35.7%)	‐	40 (37.4%)
Patient already tried alternative medication to NS β‐blocker	10 (18.5%)	6 (27.3%)	4 (28.6%)	3 (17.6%)	23 (21.5%)
No alternative available for NS β‐blocker, β‐blocker seemed the best option	15 (27.8%)	1 (4.5%)	4 (28.6%)	5 (29.4%)	25 (23.4%)
Prescriber stated that there was no actual lung disease at start of the NS β‐blocker	2 (3.7%)	‐	‐	3 (17.6%)	5 (4.7%)
No initiation, but repeating an earlier prescription of another prescriber	4 (7.4%	‐	‐	1 (5.9%)	5 (4.7%)
NS β‐blocker only for short term use	2 (3.7%)	‐	‐	‐	2 (1.9%)
Other reasons	‐	1 (4.5%)	1 (7.1%)	5 (29.4%)	7 (6.5%)

Abbreviations: CA, cardiologist; GP, general practitioner; OP, ophthalmologist; OS, other specialist.

Seventy‐seven (72.0%) of the 107 prescribers would have reconsidered the use of the NS β‐blocker when the patient had suffered from exacerbations after the start of the NS β‐blocker. Stratification for the different disciplines showed 46 GPs (85%), 14 ophthalmologists (63.5%), 7 cardiologists (50%), and 10 other initiators (58.5%) that would prescribe alternative medication in case of an exacerbation (*P* = .017).

Forty‐six initiators (30.0%) mentioned not to have been aware of the lung disease when prescribing the NS β‐blocker with the highest proportions in ophthalmologists (51.1%) and cardiologists (41.2%) and compared to GPs (15.6%, *P* = .000). Most frequently mentioned reasons for this were incomplete patient records (n = 18) and the absence of asthma or COPD at the moment of prescription (n = 18). Four doctors (2 cardiologists and 2 ophthalmologists) declared that the lung disease was not part of their specialism. Fifteen of the 46 initiators (32.6%) declared that they would have chosen an alternative medication if they had been aware of the drug‐disease interaction.

### 3.2 Surveillance signals in the community pharmacy

Two hundred ninety‐nine medication surveillance signals at the start of the NS β‐blocker in all participating pharmacies were checked. Patient characteristics are shown in Table 1. The mean patient age was 69.5 years, and 133 (44.5%) were men. A total of 122 (40.8%) patients were examined with asthma, 106 (35.5%) with COPD, and 51 (17.1%) had both symptoms of asthma and COPD. Patients used mostly ocular timolol (39.8%), and the oral preparations propranolol (30.8%) and carvedilol (15.1%).

**Figure 2:**
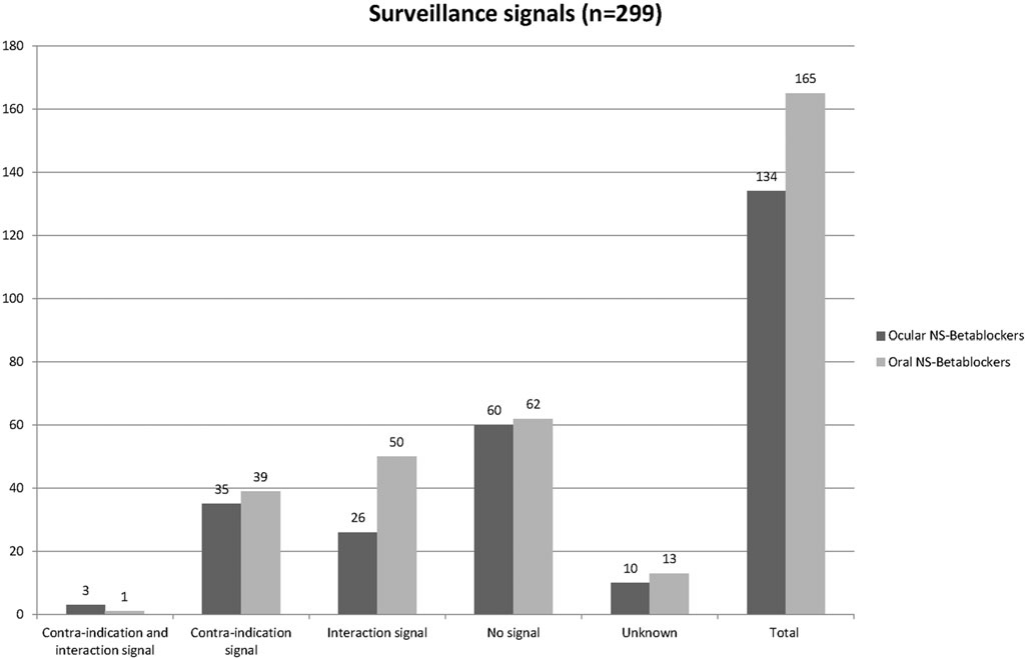
Medication surveillance signals, generated by the pharmacy information system. NS, non‐selective

**Figure 3:**
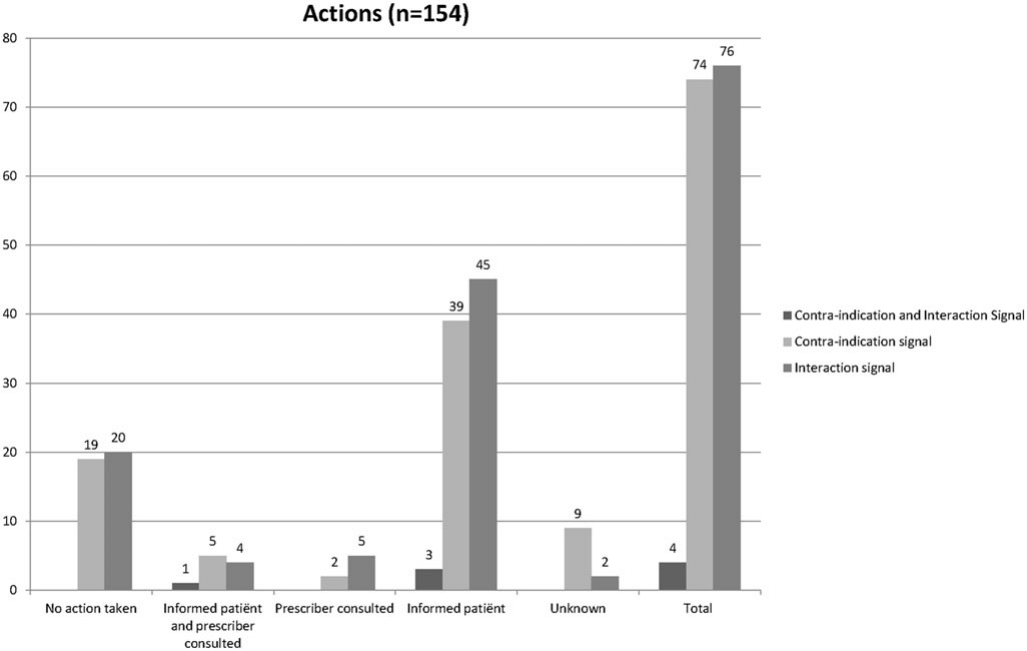
Pharmacist interventions on a medication surveillance signal

In 122 cases (40.8%), the pharmacy information system did not generate any medication surveillance signal. In 154 cases, the system generated a contraindication signal (n = 74), an interaction signal (n = 76), or both (n = 4) (Figure 2). For the cases without any signal generated by the system, the lung medication mainly did not appear as actual medication at initiating the NS β‐blocker (n = 94) or medication surveillance signals were not used in the pharmacy during the first dispensing (n = 20). For 23 patients, pharmacists could not recall the handling process of the first prescription, mainly due to the lack of digital archiving of handling the surveillance signals in the past. Processing of the medication surveillance signals is shown in Figure 3. In most cases, the patient was informed about the possibility of increased respiratory symptoms (n = 87).

## 4 DISCUSSION

Our study showed that two‐thirds of the interviewed prescribers of oral and ocular NS β‐blockers prescribed this medication to asthma or COPD patients deliberately, considering the drug‐disease interaction not to be relevant. During the interviews, ophthalmologists argued that they had never seen exacerbations in daily practice, which is not surprisingly as exacerbations are mainly treated by GPs or lung specialist. One‐third of the prescribers was simply not aware of the presence of co‐morbidity conditions.

Raising awareness and alertness of prescribers for the presences of co‐morbidities that might give rise for different choices in drug treatment because of potential contraindications could help to improve the prescribing of NS β‐blockers to patients with lung diseases. Additionally, the role of community pharmacists in signalling possible problems due to drugs prescribed by different medical specialists should be emphasized.

Many ophthalmologists held the view that the NS β‐blockers can be prescribed despite asthma or COPD. However, despite the relatively low dosage, ophthalmic timolol is more akin to intravenous delivery than to oral dosing, in terms of systemic bioavailability, plasma kinetics, and cardiopulmonary effects.^15^, ^31^ Approximately 80% of an ocular administered drop enters the systemic circulation through the highly vascular nasal mucosa, without the benefit of the first‐pass metabolism by the liver.^6^, ^32^ Although nasolacrimal occlusion, if applied properly, was shown to be a useful technique to reduce this absorption, it does not completely prevent systemic adverse effects.^33^, ^34^, ^35^ A recent meta‐analysis showed that ocular dosing with NS β‐blockers significantly affected lung function and increased asthma morbidity.^14^ So there is evidence opposed to the view that the contraindication is irrelevant, and this result is reflected by recommendations from recent studies^14^, ^15^, ^36^ and the national and international guidelines on the use of NS β‐blockers in patients with lung diseases.^2^, ^3^ In a recent study, GPs indicated that different factors (eg, multiple health care professionals involved) contributed to a complex environment, which could result in potentially inappropriate prescribing.^37^

The development of clinical guidelines only makes sense, if the care is actually implemented in health care practice. The use of guidelines in daily practice should be evaluated regularly, to identify room for improvement in practice and, if necessary, in the guidelines.^38^ Our study showed that the implementation of the recommendation to avoid NS β‐blockers in patients with lung diseases had led to the general process of medication surveillance signals into the pharmacy information system, but that this computerized signals clearly did not necessarily result in actions by the pharmacists. In 39 of the 154 cases, the pharmacists ignored the signal and decided not to take any action (eg, consulting the prescriber or adjustment of the therapy). This is consistent with other research on decision support systems in pharmacies.^39^

In almost half of the cases studied, pharmacists did not receive any medication surveillance signal in daily practice. Interaction signals were less generated for ocular—than for oral NS β‐blockers (26 versus 50 times, respectively). A precondition for an interaction signal is the concomitant use of NS β‐blockers and inhalation medication (including β‐agonists and ICS). Calculated drug use periods from dispensing information may not correspond with actual patients' drug use. This may be especially the case for both eye drops or inhalation medication, as the daily dose may be accustomed due to symptoms or to the concomitant use of different inhalers or eye drop bottles. In daily practice, this kind of medication history errors is common.^40^, ^41^ Combination of pharmacy records and patient counselling could result in an up‐to‐date and complete medication overview including current medication use and all medication allergies or intolerances, so this can be an important assignment for each community pharmacist.

This study has several limitations. Patient selection and sampling by community pharmacists might have been influenced by individual preferences. Additionally, patients with treatments initiated in hospital might have been missed. Besides, as the prescribers were interviewed about prescribed NS β‐blockers in the past, the possibility of recall bias cannot be excluded. For example, this could have led to more socially desirable answers in case of an incomplete dossier, like “the patient has tried alternative medication” or “there was not any lung disease yet.” Participating pharmacists were unable to assess whether the prescribers were in the possession of a complete dossier. Furthermore, all selected patients were chronic users of NS β‐blockers, which could have led to survival bias; long‐term treatment is more likely to occur in patients who tolerate acute exposure, whereas patients with side effects directly after the start with NS β‐blockers are more likely to switch to alternative medication or stop the treatment.^14^

Further research is needed to estimate whether NS β‐blockers may trigger the development of symptoms, whether this depends on different dose levels of NS β‐blockers or the duration of therapy, possible switching of dose levels in the past, or on specific co‐medication or co‐morbidities. Evaluation of clinical outcomes is part of the implementation process, and this is an important topic for future research.

In conclusion, this study showed that, in contrast to prevailing guidelines, part of the interviewed prescribers did not assume NS β‐blockers to be contraindicated in the selected patients with asthma or COPD. There is no evidence to support this statement, and although the recommendation of avoiding NS β‐blockers is implemented in the medication surveillance system in the pharmacy, pharmacists still need to be aware of the evidence‐based clinical background of the generated signals and the importance of appropriate handling.

Further research is needed to evaluate to which extent the mentioned considerations are legitimate and to estimate clinical outcomes in patients with asthma and COPD, which are (deliberately or unintentionally) treated with NS β‐blockers.

## CONFLICT OF INTEREST

The authors declare no conflict of interest.

## ETHICAL APPROVAL

No ethical approval required for this study.

## Supporting information

Supporting info item
