# Supplementary material for: Considerations of prescribers and pharmacists for the use of non‐selective β‐blockers in asthma and COPD patients: An explorative study
Source: J Eval Clin Pract. 2018 Jan 10;24(2):396–402. doi: 10.1111/jep.12869 (PMC5901013; doi:10.1111/jep.12869)
Supplement: Supplementary file 1 — Supporting info item [file JEP-24-396-s001.docx]

# Appendix A: Semi-structured format for the interview with prescribers

### Central question: Was the prescriber aware of the airway disease at the moment of prescribing the β-blocker?

***Yes:***

- *If so; why did he or she prescribe the non-selective β-blocker despite the contra-indication?*
  - - *Contra-indication not relevant in prescribers’ opinion*
    - *Other medication already tried, but it didn’t work*
    - *There was no alternative*
    - *Other reason, ………*
  - *If the patient would suffer from exacerbations after the start of the* non-selective β-blocker, would the prescriber reconsider his/her choice*?*
    - - *If so, what would the choice be for this patient?*
      - *If not, what are the reasons?*

***No:***

- *If not; what was the possible reason for not knowing the contra-indication in his/her opinion?*
- *Would the prescriber have chosen an alternative medicine when the contra-indication asthma or COPD was known?*
  - - *If so: which alternative would be chosen?*
    - *If not: what are his/her reasons?*
      - *Contra-indication not relevant in prescribers’ opinion*
      - *Other medication already tried, but it didn’t work*
      - *There was no other choice*
      - *Other reason, ………*
- *If the patient would suffer from exacerbations after the start of the* non-selective β-blocker, would the prescriber reconsider his/her choice*?*
  - - *If so: what would the choice be for this patient?*
    - *If not: what are the reasons?*
